# Evaluation of tip fold over in 1,575 cochlear implant electrode arrays

**DOI:** 10.3389/fneur.2026.1976958

**Published:** 2026-09-11

**Authors:** Givi Magradze, Jonas Schröfel, Leonie Fries, Cansel Yildiz, Antje Aschendorff, Susan Arndt, R.L. Beck, M.C. Ketterer

**Affiliations:** Department of Otorhinolaryngology, Medical Center – University of Freiburg, Faculty of Medicine, University of Freiburg, Freiburg, Germany

**Keywords:** cochlear implant, electrode array design, Perimodiolar array, straight electrode array, tip fold over

## Abstract

**Objective:**

Cochlear implant (CI) surgery aims to achieve atraumatic electrode array insertion to preserve residual hearing and optimize outcomes. Tip fold-over, a mechanical complication during insertion, can compromise stimulation effectiveness and may require reinsertion or revision surgery. This study investigates the incidence, influencing factors, and diagnostic approaches for tip fold-over across a large patient cohort implanted with various CI electrode array designs.

**Methods:**

We conducted a retrospective analysis of adult patients who underwent CI surgery between 2013 and 2024 at the Department of Otorhinolaryngology–Head and Neck Surgery, Medical Center–University of Freiburg. Included electrode arrays were from Cochlear™ (Slim Straight [SSA], Slim Modiolar [SMA], Contour Advance [CA]), MED-EL (FlexSoft, Flex28, Flex26, Flex24), and Advanced Bionics (SlimJ, Mid Scala [MS]). Detailed reviews of surgical reports, intraoperative electrophysiological data, and postoperative imaging—evaluated independently by at least two reviewers—were performed. Diagnostic imaging modalities included cone beam computed and digital volume tomography (DVT).

**Results:**

Among 1,575 implanted ears, 13 cases of tip fold-over were detected, yielding an overall incidence of 0.83%. Notably, 10 of these occurred with precurved, perimodiolar arrays (SMA and CA, Cochlear™; MS, Advanced Bionics), while only 3 were found in straight lateral wall designs (SSA, Cochlear™). No tip fold-over events were observed with any MED-EL electrode arrays or with the SlimJ electrode array from Advanced Bionics. Intraoperative Transimpedance matrix (TIM) analysis and intraoperative X-ray reliably detected tip fold-over in cases with normal anatomy.

**Conclusion:**

Electrode array design significantly influences tip fold-over risk. A potentially higher incidence has been observed with perimodiolar electrode arrays compared with flexible straight arrays. The importance of intraoperative diagnostics is underscored in this study. TIM analysis and intraoperative X-ray imaging proved effective in detecting tip fold-over events in real-time, allowing for immediate corrective action and avoiding revision surgeries. This large-scale retrospective study confirms that tip fold-over remains a rare but significant complication in CI surgery, with a higher prevalence in precurved perimodiolar electrode arrays. This study emphasizes the need for ongoing vigilance across all CI systems.

## Introduction

An increasing number of cochlear implant (CI) electrode array designs have been developed to achieve the goal of atraumatic insertion (1). Due to individual variability in cochlear size (2–4), CI manufacturers offer a range of electrode array designs with varying diameters and lengths (5, 6). Furthermore, two main principles of stimulation are represented based on the position of the electrode array: perimodiolar electrode arrays (PM) with pre-curved designs aiming to stimulate near the modiolus (1, 7), while lateral wall electrode arrays are designed to be positioned farther from the modiolus along the lateral wall to minimize trauma within the cochlea (8, 9, 53). The most common form of cochlear trauma due to electrode insertion is scalar translocation, i.e., dislocation from one scala to another (2, 10). The goal is still a complete insertion of the electrode array into the scala tympani (11, 12, 54), as this provides the best conditions for hearing rehabilitation (13). The risk of insertion trauma has decreased over time due to greater surgical experience (14) and the development of less traumatic electrode arrays (1, 15–18, 52). A complete scala tympani insertion also plays an important role in preserving residual hearing (19) and may influence the scalar position of the electrode array if reinsertion is required in the future (16, 18, 20).

As described by Dhanasingh and Jolly (8), another potential trauma during electrode array insertion is tip fold-over, where the tip folds back on itself, potentially causing cochlear trauma and mis-stimulation and consequently a reduction of insertion depth. This complication has been reported in multiple studies before for the Slim Modiolar electrode array (SMA; models 532/632), and the Contour Advance electrode array (CA; models CI24RECA, CI412/512/612) from Cochlear Limited (NSW, Australia) (8, 21–31). Furthermore, other studies have reported the occurrence of tip folds in MED-EL (Innsbruck, Austria) electrode arrays (32) and in electrode arrays from Advanced Bionics (Valencia, United States) (31).

The aim of this study is to evaluate the occurrence of tip fold-over in a large patient cohort, to assess whether individual or institutional learning curves can reduce the incidence of this complication, and to determine whether intraoperative versus postoperative diagnostics—such as electrophysiology, Transimpedance matrix (TIM) analysis, and imaging—are effective in identifying tip fold-over and enabling prompt reinsertion, thereby avoiding the need for revision surgery.

## Methods

This is a retrospective analysis of adult patients who received CIs between 2013 and 2024 at the Department of Otorhinolaryngology–Head and Neck Surgery, Medical Center–University of Freiburg. Patients implanted with devices from the following manufacturers and electrode arrays were included: the Slim Straight electrode array (SSA; models 422/522/622), the Slim Modiolar electrode array (SMA; models 532/632), and the Contour Advance electrode array (CA; models CI24RECA, CI412/512/612) from Cochlear Limited (NSW, Australia); the MED-EL FlexSoft, Flex28, Flex26 and Flex24 arrays from MED-EL (Innsbruck, Austria); and the SlimJ and MidScala (MS) arrays from Advanced Bionics (Valencia, United States).

A thorough review was conducted of all surgical reports, intraoperative measurements, and follow-up documentation including pre- and postoperative residual hearing. Additionally, postoperative imaging was re-evaluated by at least two independent authors of this study. Imaging modalities included cone beam computed tomography (CBCT) with a DynaCT-equipped Axiom Artis dTA angiography unit (Siemens Co., Erlangen, Germany) until 2022, and digital volume tomography (DVT) using the NewTom 5G/GXL system (Hillus Medical Engineering KG) from 2022 onward, as previously described by our research group [(1, 2, 11, 17, 33); (5, 20, 22, 52)]. We excluded patients with clear signs of cochlear fibrosis or abnormal anatomy, such as incomplete partition type III, as previous studies have described differing insertion conditions in these cases (34). All included ears demonstrated morphological characteristics within the range previously described by Ketterer et al. (2), with distances A and B assessed according to the definitions established by Escudé et al. (35).

This study was approved by the Freiburg medical center Ethics Committee (approval number: 406/19; amendment numbers: 240275), and is registered in the Freiburg Clinical Trials Register (FRKS number: FRKS006332) and the German Clinical Trials Register (DRKS number: DRKS00037881).

## Results

### Distribution and influence of electrode array design

**Figure 1:**
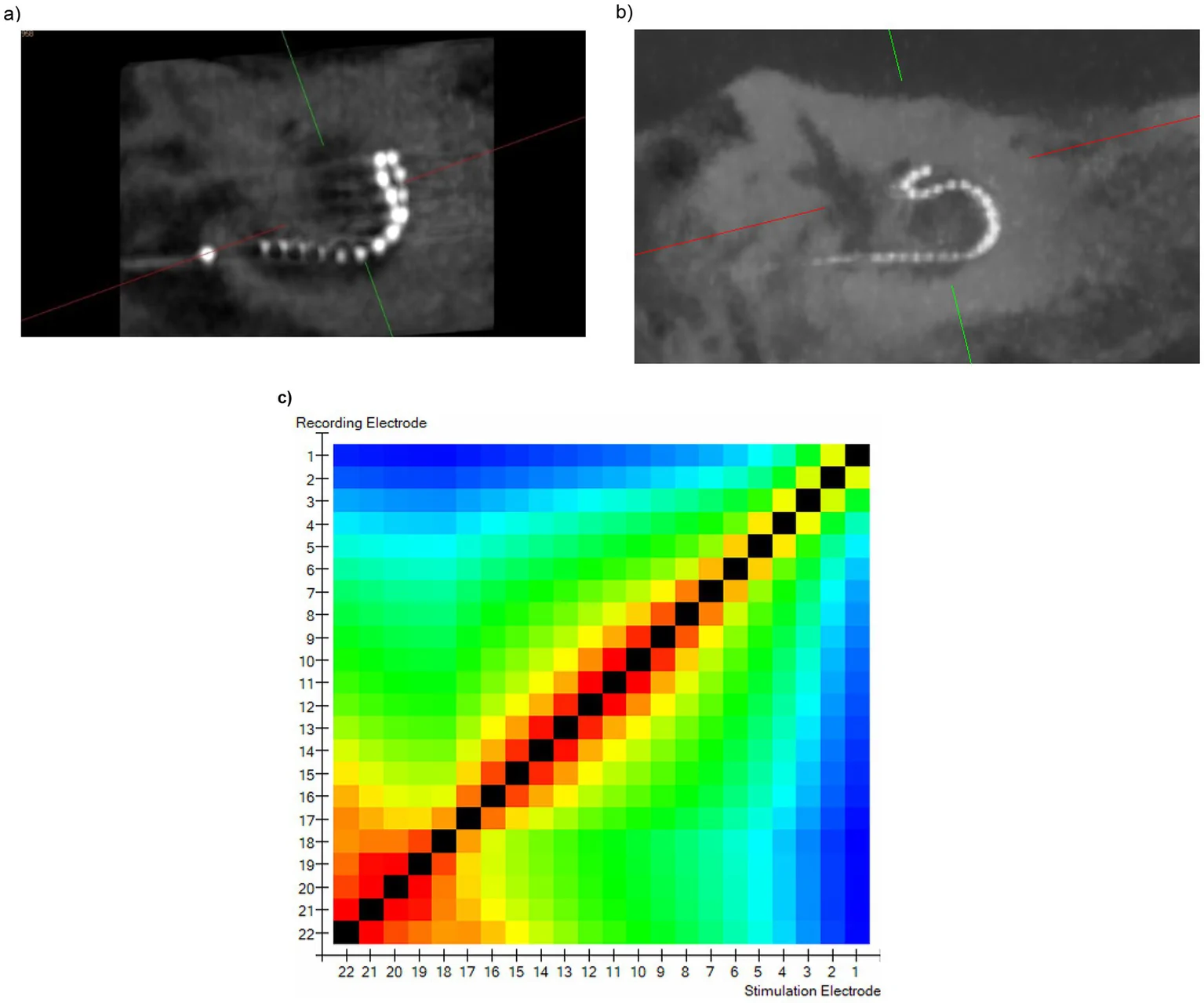
Postoperative cone-beam computed tomography demonstrating electrode array tip fold-over **(a)** Example of a tip fold-over involving a Mid-Scala (MS) array from Advanced Bionics **(b)** Example of a tip fold-over involving a Slim Straight Array (SSA) from Cochlear™ **(c)** Corresponding abnormal intraoperative TIM measurement, which clearly detected the tip fold of the SSA **(b)**.

After applying the inclusion criteria, a total of 1,575 implanted ears were analyzed. These procedures were performed between 2013 and 2024 at the Department of Otorhinolaryngology–Head and Neck Surgery, Medical Center–University of Freiburg. Devices from three manufacturers were included in the analysis: Cochlear™, MED-EL, and Advanced Bionics (see Table 1). Within this cohort, 13 cases of tip fold-over were identified, corresponding to an overall tip fold-over incidence rate of 0.83%. The majority of these events (10 cases) were associated with precurved, perimodiolar electrodes, specifically the SMA, CA, and one case in the MS array (see Figure 1). This represents a tip fold-over rate of 1.59% among the 629 precurved perimodiolar electrode arrays implanted. Focusing on the Advanced Bionics MS electrode array specifically, only one tip fold-over event was observed among 114 implanted ears, yielding a tip fold-over rate of approximately 0.88%.

**Table 1:** Distribution table of the included and analyzed manufacturers and electrode arrays (*n* total = 1,575).

Manufacturer	Electrode array	*n* total
Cochlear TM	SSA	553
	SMA	159
	CA	356
MED EL	Flex24	31
	Flex26	76
	Flex28	170
	FlexSoft	4
Advanced Bionics	MS	114
	SlimJ	112

In contrast, only 3 cases of tip fold-over were detected among the 946 lateral wall (straight) electrode arrays, which include the Cochlear™ SSA, MED-EL Flex series, and Advanced Bionics SlimJ. The incidence rate in this group was significantly lower, at 0.32%. All three tip fold-over cases in this group involved lateral wall SSA arrays, corresponding to an incidence of approximately 0.54% (3 out of 553). No tip fold-over cases were detected with the Advanced Bionics SlimJ or any of the included MED-EL electrode arrays. Of the 13 tip fold-over events, 11 occurred during primary implantation, and 2 occurred during reimplantation procedures for device failure. Residual hearing, expressed as PTA-4, was present preoperatively in 8 of the 13 affected cases. Postoperatively, residual hearing deteriorated in 2 of these cases, while the remaining 5 cases lost residual hearing completely (see Table 2).

**Table 2:** Clinical, audiological, and surgical characteristics of the 13 cases with tip fold-over.

No.	Age	Year of surgery	Reimplantation	Otogenic diagnosis	Tip fold-over findings	Manufacturer	Electrode	New Implant required	Second electrode	Transimpedance matrix measurement (TIM)	X-ray	Preoperative residual hearing (PTA-4)	Postoperative residual hearing (PTA-4)	Surgeon experience
1	55	2014	No	PHL	TFP	AB	MS	No	0	NT	NP	65 dB	115 dB	JS
2	60	2015	No	PHL	TFP	Cochlear	CI 512	No	0	NT	NP	0	0	SS
3	41	2015	No	PHL	TFP	Cochlear	CI 532	No	0	NT	P	110 dB	0	SS
4	27	2015	Yes	DF	TFI	Cochlear	CI 512	No	0	NT	NP	0	0	SS
5	77	2016	Yes	DF	TFI	Cochlear	CI 512	No	0	NT	NP	0	0	SS
6	46	2020	No	PHL	TFI	Cochlear	CI 632	Yes	CI 612	TAN	P	100 dB	0	SS
7	56	2021	No	PHL	TFP	Cochlear	CI 622	No	0	TAN	N	100 dB	100 dB	SS
8	72	2021	No	PHL	TFP	Cochlear	CI 622	No	0	TAN	N	95 dB	0	SS
9	57	2022	No	PHL	TFI	Cochlear	CI 622	No	0	TAN	P	110 dB	0	SS
10	55	2022	No	PHL	TFI	Cochlear	CI 632	No	0	TAN	N	80 dB	110 dB	SS
11	73	2023	No	PHL	TFI	Cochlear	CI 632	No	0	TAN	N	0	0	JS
12	54	2023	No	PHL	TFI	Cochlear	CI 632	No	0	TAN	N	75 dB	0	SS
13	31	2023	No	PHL	TFI	Cochlear	CI 632	No	0	TAN	N	0	0	JS

The table lists age at surgery, year of implantation, reimplantation status, otogenic diagnosis, mode of tip fold-over detection, manufacturer, electrode type, requirement for a new implant, secondary electrode used (if applicable), results of transimpedance matrix measurement (TIM), radiological findings (X-ray), pre- and postoperative residual hearing expressed as PTA-4 (pure tone average at 0.5, 1, 2, and 4 kHz), and surgeon experience.

PHL, Progressive Hearing Loss; DF, Device Failure; TIM, Transimpedance Matrix Measurement; AB, Advanced Bionics; MS, Mid-Scala; JS, Junior Surgeon; SS, Senior Surgeon; N, Normal finding; P, Pathological finding; NP, No X-ray performed; NT, Prior to the implementation of TIM; TAN, First TIM abnormal, post-reimplantation TIM normal; TFI, Tip fold-over detected intraoperatively; TFP, Tip fold-over detected postoperatively.

### Influence of intraoperative measurements

Intraoperative tip fold-over detection was documented in 8 cases (61.5%), while postoperative detection occurred in 5 cases (38.5%) and was detected with either CBCT or DVT (see Table 2). Intraoperatively detected tip fold-overs were managed within the same surgical session. In one case, repeated attempts to reinsert the SMA failed to correct the fold-over, necessitating the use of a second implant (CA), which was successfully inserted.

We performed TIM constantly in Cochlear TM electrode arrays following the introduction with surgical routine measurements in June 2019. TIM measurements were performed intraoperatively in 8 cases, all showing abnormal findings consistent with tip fold-over. The remaining cases occurred prior to the implementation of TIM. Both reimplantation procedures (see Table 2) were performed prior to the implementation of TIM.

Final radiological evaluation was performed with modified transorbital intraoperative X-ray imaging. Three examinations revealed abnormalities consistent with tip fold-over. In one notable case (see Table 2, case nr. 3), an abnormal intraoperative X-ray finding led to reinsertion of the electrode array, followed by a normal X-ray result. However, a postoperative cone beam CT demonstrated a tip fold-over. Importantly, this case occurred before the implementation of TIM measurements, which were therefore not available for intraoperative guidance. A crucial aspect to emphasize is the timing of intraoperative radiological imaging. Except for two cases, X-ray examinations were routinely performed at the end of surgery. When abnormal TIM findings suggested a possible tip fold-over, the electrode array was first removed and reinserted. After normalization of the TIM measurements, intraoperative X-ray imaging was performed at the end of the procedure to confirm the final electrode array position. This workflow allowed TIM to serve as the primary intraoperative screening tool for detecting abnormal insertion patterns while avoiding unnecessary additional radiation exposure from repeated intraoperative imaging. In the two exceptional cases, X-ray imaging was performed immediately after abnormal TIM findings, which confirmed tip fold-over and guided subsequent successful reinsertion of the electrode array. Detailed demographic, audiological, and surgical data for all 13 tip fold-over cases are provided in Table 2.

### Influence of surgical experience

**Figure 2:**
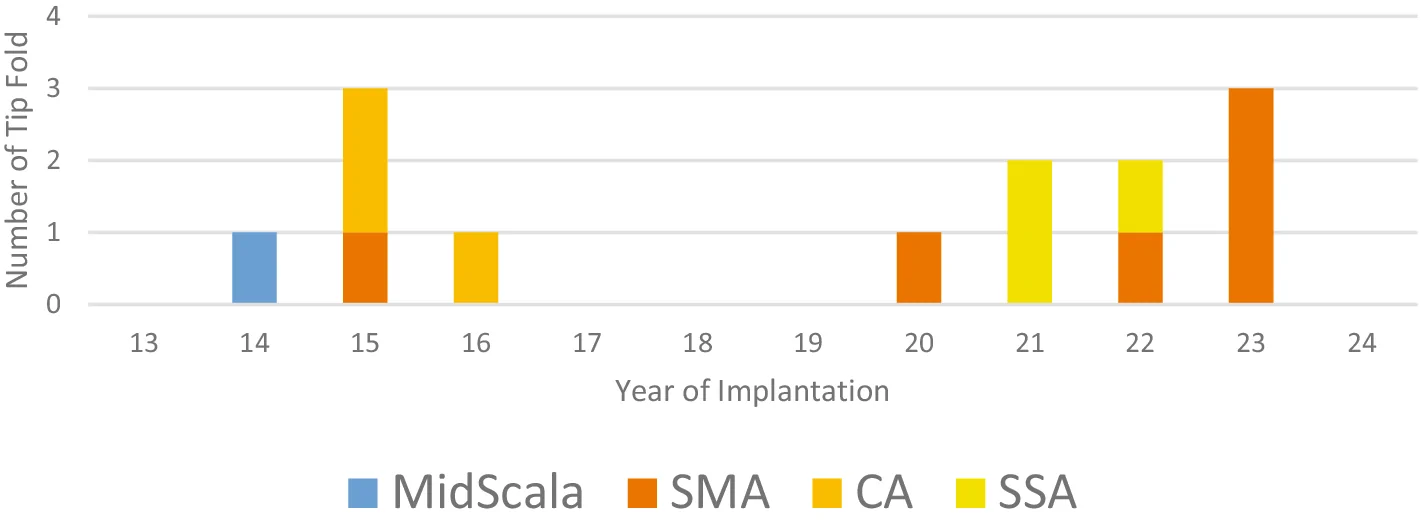
Annual distribution of tip fold-over cases according to electrode array type. The x-axis represents the year of implantation and the y-axis indicates the number of tip fold-over events identified in that year. Colors correspond to the implanted electrode array type: Mid-Scala (MS), Slim Modiolar Array (SMA), Contour Advance (CA), and Slim Straight Array (SSA).

**Figure 3:**
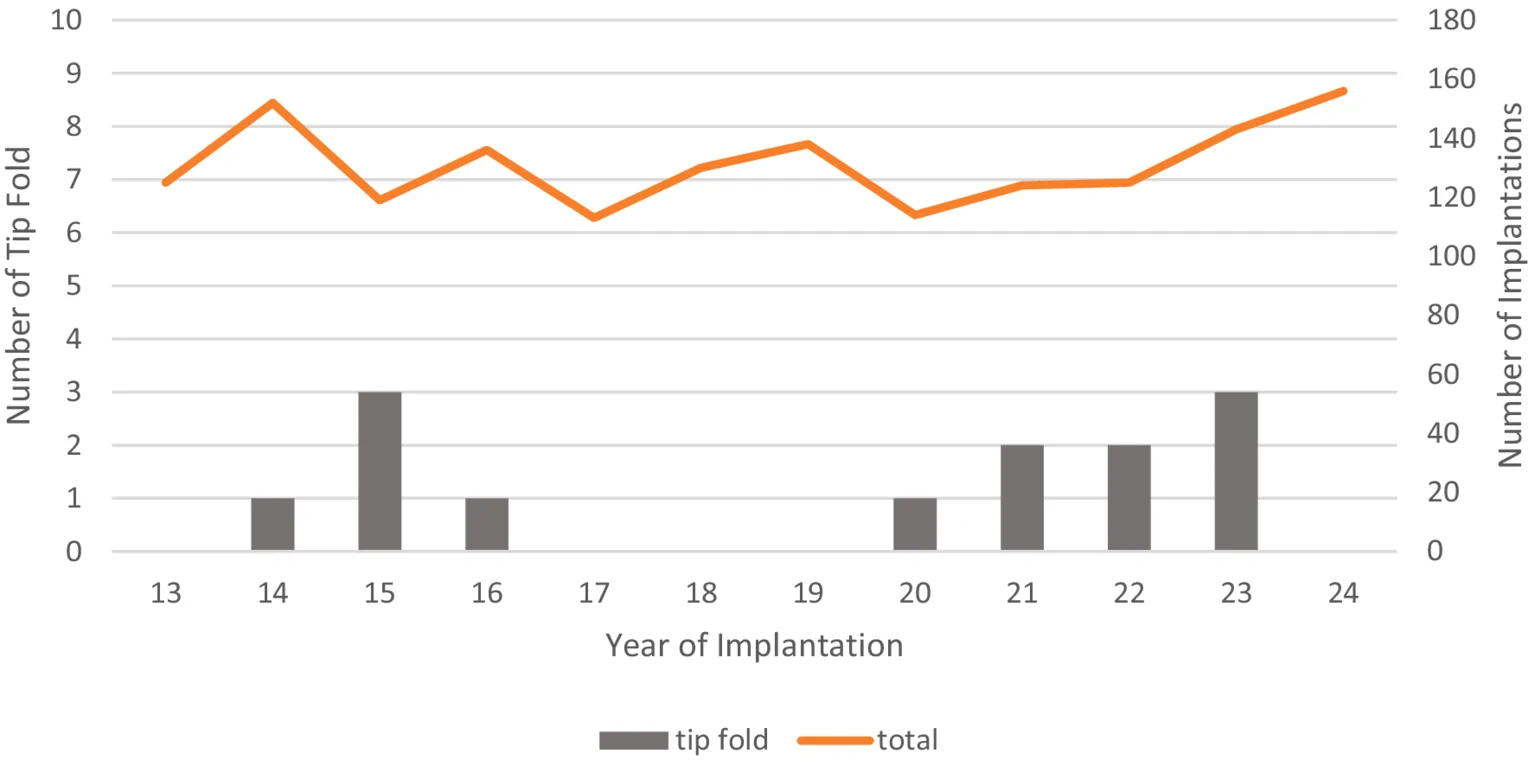
Annual distribution of total implantations and tip fold-over cases. The *x*-axis represents the year of implantation. The left *y*-axis shows the total number of tip fold-over events per year (all electrode types combined), while the right *y*-axis indicates the total number of cochlear implantations in adult patients performed annually.

We additionally examined the potential influence of surgeon experience. Among the 13 cases involving tip fold-over, 11 procedures were performed by a senior surgeon and 2 by a junior surgeon. This distribution likely reflects the greater overall number of procedures conducted by senior surgeons within the cohort rather than an effect of surgical expertise per se. Specifically, Senior Surgeon A performed 703 cochlear implantations with 4 tip fold-over events (0.57%), Senior Surgeon B performed 554 implantations with 6 tip fold-over events (1.08%), and Senior Surgeon C performed 74 implantations with 1 tip fold-over event (1.35%). Among junior surgeons, Surgeon D performed 45 implantations with 1 tip fold-over event (2.22%), and Surgeon E performed 56 implantations with 1 tip fold-over event (1.79%), whereas the remaining five junior surgeons collectively performed 143 implantations without any tip fold-over events. A senior surgeon was defined as a surgeon who had more than 5 years of surgical CI experience. An analysis of the annual distribution of tip fold-over cases showed an initial decline following the first occurrences in 2014 and 2015. No events were documented between 2017 and 2019. From 2020 onward, however, a gradual increase in cases was noted, reaching a peak in 2023 (Figures 2 and 3). These findings suggest that tip fold-over remains a sporadic yet persistent complication over time.

## Discussion

### Distribution and influence of electrode array design

Tip fold-over is a well-known complication in CI surgery and is particularly influenced by electrode array design. Previous studies have shown that precurved, perimodiolar electrode arrays are associated with a higher incidence of tip fold-over compared to straight, lateral wall electrode arrays (26). The present study confirms these findings in one of the largest cohorts reported to date. We detected 13 cases of tip fold-over in 1575 implanted ears (0.83%): 10 occurred in perimodiolar electrode arrays and 3 in the straight electrode array of Cochlear (SSA). No tip fold-over events were observed in any of the evaluated MED-EL electrode arrays or in the SlimJ array of Advanced Bionics.

In a large retrospective review, Högerle et al. (26) reported a tip fold-over rate of approximately 5.3% for perimodiolar electrode arrays, compared with 0.5–1% for straight lateral wall electrodes. In our study, lower incidence rates were observed, with 1.59% in perimodiolar electrodes and 0.32% in straight electrode arrays. The increased risk associated with perimodiolar electrode arrays has been attributed to their inherent shape and mechanical properties, which can predispose the tip to buckle or rotate during insertion, particularly in anatomically challenging cochleae (36). Their fixed curling geometry may interact unpredictably with the variable anatomy of the cochlear modiolus, resulting in mechanical buckling or rotation of the array tip during insertion.

Notably, the tip fold-over rate in our study was not uniform across all three included precurved electrode arrays (SMA, CA, and MS). The MS array of Advanced Bionics exhibited only one tip fold-over event in 114 cases (0.88%).

A large retrospective review of 1,722 CIs—including perimodiolar, lateral wall, and MS electrode arrays—found an overall tip fold-over rate of 0.87% (24). While a temporal bone study evaluating the Advanced Bionics MS electrode demonstrated reliable, atraumatic insertions without any observed tip fold-over events in 20 insertions (37), a clinical study described the occurrence of tip fold for the MS electrode array before (31). In summary, tip fold-over is a recognized complication that appears to occur more frequently with precurved and perimodiolar electrode arrays.

Tip fold-over has been linked to transient increases in intracochlear pressure, particularly when the electrode twists or rotates (36). Additionally, Cochlear duct length has been identified as a predisposing factor, with longer cochlear ducts increasing the risk—especially for proximal fold-over events in slim modiolar electrodes (38). In contrast, straight lateral wall arrays are more flexible and conform to the cochlear curvature, resulting in a much lower propensity for tip fold-over (6, 39, 40). Overall, precurved perimodiolar arrays carry a substantially higher risk of tip fold-over than straight lateral wall arrays, and this difference has been consistently demonstrated across multiple device manufacturers and clinical settings (6, 24, 26, 38, 39).

### Influence of intraoperative measurements

Intraoperative detection of tip fold-over is essential, as unrecognized tip fold-over can result in poor speech perception outcomes and other postoperative complications. While fluoroscopy has traditionally been used for this purpose, newer techniques such as TIM and capacitive sensing systems have demonstrated higher sensitivity and reliability for detecting tip fold-over events (41–43).

In our cohort, intraoperative TIM measurements were performed in 8 of the 13 cases with tip fold-over, and all 8 showed abnormal findings consistent with tip fold-over. This underscores the effectiveness of TIM in providing immediate intraoperative feedback, enabling correction before wound closure. Since June 2019, TIM has been routinely applied in all Cochlear TM CI procedures at our center, allowing for prompt detection and management of tip fold-over events during the same surgical session. Although TIM has demonstrated high sensitivity for detecting tip fold-over, false-positive findings may occur; therefore, TIM results should always be interpreted in conjunction with intraoperative imaging and the clinical context (44, 45). Especially in reimplantations due to possible fibrotic changes (16, 18, 20) and in malformations, e.g., incomplete partition type III (34).

Radiological evaluation further highlighted the limitations of conventional X-ray imaging in detecting fold-over. One particularly notable case in this study occurred before the implementation of TIM: an abnormal intraoperative X-ray prompted reinsertion. Following reinsertion, X-ray imaging showed no evidence of tip fold-over, yet postoperative cone-beam computed tomography (CBCT) still demonstrated a tip fold-over (case number 3; see Table 2).

Furthermore, in another patient (case number 8; see Table 2), TIM indicated a possible tip fold-over (Figure 1c). An X-ray was performed immediately afterward but showed no signs of tip fold-over, so the electrode array was left in place. However, 2 days later, a cone-beam computed tomography (CBCT) scan (Figure 1b) clearly demonstrated a tip fold-over, leading to revision surgery. These findings highlight the potential for false-negative results when relying solely on modified transorbital X-ray imaging, as a tip fold-over may not be detectable depending on its orientation relative to the X-ray projection.

Our findings support previous reports indicating that intraoperative electrophysiological monitoring is especially important for slim modiolar and other precurved electrode arrays, where tip fold-over occurs more frequently and may be missed by standard X-ray imaging alone (46, 47). Although intraoperative imaging such as CBCT or high-resolution fluoroscopy is less frequently required, it remains valuable in cases of challenging cochlear anatomy or when resistance is encountered during insertion (26, 34, 39).

### Influence of surgical experience

In our cohort, of the 13 cases with tip fold-over, 11 procedures were performed by senior surgeons and 2 by junior surgeons. However, this distribution must be interpreted in the context of the substantially higher procedural volume among senior surgeons. Indeed, the calculated tip fold-over rates per surgeon remained low across all experience levels, ranging from 0.57 to 1.35% among senior surgeons and from 1.79 to 2.22% among junior surgeons demonstrating both individual and in-house learning curves. Furthermore, no tip fold-over events occurred in the remaining five junior surgeons, despite a combined total of 143 cochlear implant procedures. These findings suggest that tip fold-over may occur even in the hands of highly experienced CI surgeons and is likely influenced more strongly by electrode design, insertion mechanics, and individual cochlear anatomy than by surgical experience alone. Perimodiolar, precurved electrode arrays are designed with a predefined curvature and are typically inserted using a stylet or a sheath for the SMA. Upon removal of the stylet, the electrode array assumes its preformed shape and is intended to position itself close to the modiolus. However, this mechanism carries an inherent risk: if the electrode is released too early or too late, or if the individual cochlear anatomy—such as an increased cochlear duct length —does not match the fixed curvature of the electrode, the tip may buckle, resulting in a tip fold-over (38, 40, 48). It should also be noted that the SMA and the MS must be inserted using AOS technique, whereas the CA does not necessarily have to be inserted using AOS technique.

In contrast, straight lateral wall electrode arrays from MED-EL are not precurved but are highly flexible and passively follow the lateral wall of the cochlea during insertion. These arrays do not require a stylet and lack a predefined curvature that could conflict with individual cochlear anatomy. As a result, the risk of tip fold-over is considered to be very low in these electrode designs (26).

Meticulous, slow, and controlled insertion remains the most effective strategy to minimize tip fold-over, although the optimal technique varies according to the type of electrode array. For straight lateral wall arrays, which inherently carry a low risk of tip fold-over, a slow, continuous insertion along the lateral wall—ideally via the round window or extended round window—is recommended. Careful handling to avoid abrupt movements is essential to further reduce the risk of malposition (26, 39, 49, 50).

For precurved perimodiolar arrays, the insertion technique is more complex and requires additional precautions. Correct alignment to the modiolus, precise handling of the stylet in stylet-based systems, and a prolonged, controlled insertion are essential to prevent tip folding (6, 38, 51). Some studies described, that longer cochlear duct lengths (>36 mm) increase the risk of tip fold-over, emphasizing the importance of preoperative cochlear duct measurements and appropriate electrode selection (38). Intraoperative imaging or electrophysiological monitoring, such as TIM, provides immediate feedback to detect and correct malposition.

In summary, precurved perimodiolar arrays require heightened intraoperative vigilance, careful technique, and, ideally, real-time monitoring to reduce tip fold-over, while straight lateral wall arrays benefit primarily from gentle insertion under standard surgical precautions (26, 39).

In our cohort, tip fold-over occurred in 13 cases, predominantly affecting precurved perimodiolar arrays. Temporal analysis showed early cases in 2014–2015, a period without events from 2017–2019, and a resurgence from 2020, peaking in 2023. Although infrequent, these findings highlight that tip fold-over remains a sporadic but persistent complication, emphasizing the importance of meticulous intraoperative technique, surgeon experience, intraoperative measurements, and electrode array design.

Nevertheless, several limitations of this study should be acknowledged. First, this was a single-center study conducted at a large CI center. Therefore, the transferability of the surgical expertise and the reported findings to other centers, particularly smaller CI centers, may be limited. Second, the number of electrode arrays investigated varied considerably between electrode array types, which may have introduced a potential selection bias. However, nearly all currently available electrode arrays were included and analyzed, which may also be considered a strength of this study, as it provides a comprehensive overview across different electrode designs. Furthermore, the low incidence of tip fold-over limits the statistical power of the study. In particular, the occurrence of only a single tip fold-over with the MS electrode array does not allow meaningful conclusions regarding potential learning curves. Similarly, the limited number of cases does not permit robust conclusions regarding functional outcomes, such as preservation of residual hearing.

## Conclusion

The design of electrode arrays plays a critical role in influencing the risk of tip fold-over. Perimodiolar electrode arrays have been associated with a higher incidence of this complication compared to flexible straight arrays. This study highlights the pivotal importance of intraoperative diagnostics. In our cohort, intraoperative TIM analysis combined with radiographic imaging enabled reliable real-time detection of tip fold-over and immediate correction, thereby reducing the need for revision surgery. Based on these findings, TIM should be considered the primary intraoperative screening tool, with abnormal findings prompting immediate reinsertion and subsequent radiological confirmation of correct electrode placement. Advanced imaging techniques such as intraoperative cone-beam CT or digital volume tomography may provide additional diagnostic certainty in complex cases. Findings from this large-scale retrospective analysis confirm that tip fold-over, while rare, represents a clinically significant complication in CI, with a notably higher prevalence in precurved perimodiolar electrode arrays. Furthermore, this investigation also reports tip fold-over in the Advanced Bionics MS array, underscoring the necessity for continued vigilance across all cochlear implant systems. These findings should encourage all manufacturers to develop similar measurement tools to enable intraoperative diagnostics of electrode array position.

## Data Availability

The raw data supporting the conclusions of this article will be made available by the authors, without undue reservation.
